# Gynæcology

**Published:** 1880-06

**Authors:** 


### GYNÆCOLOGY.

DISPLACEMENTS OF THE UTERUS—A NEW THEORY OF THEIR MECHANISM AND TREATMENT, by Dr. G. Cowan, Kentucky, is communicated to the *American Practitioner*, 1879, and abstracted in No. 2 of *Centralblatt*, 1880. The author describes the different theories of the text-books respecting the uterine supports, and the origin of displacements by distension, relaxation, or shrinking of these supports, and concludes, on the ground of anatomical and logical investigations, that neither the vagina broad and round ligaments nor the utero-vesical nor utero-sacral ligaments are to be looked upon as the primary supports of the uterus. For it can be proved experimentally that they are not put on the stretch until displacement has already occurred, and therefore when the uterus is in its normal position they are not tight, and accordingly cannot support the uterus. All these ligaments do is to retain the uterus in its proper axis. The power of support the author now finds in the direction of the intra-abdominal pressure, which, according to him, acts as follows: The intestines glide upon three planes in an arched line downwards and backwards—namely, first, the anterior surface of the 9th, 10th, 11th, and 12th dorsal vertebræ, the 1st, 2d, and 3d sacral vertebræ, and the adjoining lateral surfaces of the abdominal cavity; second, the lower anterior and inner surface of the abdominal cavity, and the inner surface of the symphysis and ossa pubis; third, the inner surface of the rami ischii, the anterior and upper surface of the perineum, and the adjoining surfaces of the vagina and of the rectum, to which, by the coccyx and sacrum, an upwards and backwards concave direction is given. The mobile abdominal viscera glide now from the first plane downwards and forwards upon the fundus uteri and bladder; from thence the pressure passes by the second surface downwards and backwards on the third plane, and from this directly upwards and somewhat backwards upon the uterus and its ligaments, which is therefore compressed by hydrostatic pressure from before and under against the rectum, and is kept in a floating position. The uterus is to be compared to the keystone of a reversed arch, but with the thick end of the stone upwards. The bowels press now upon the arched bladder; the uterus is pear-shaped, and is held down anteriorly by the vesico-uterine ligaments, together with the ligamenta lata and the anterior and posterior duplicatures of peritoneum kept in the axis of the pelvic inlet, and can escape from this pressure directed upon it forwards and under only in the upwards and forwards direction. Were the small end of the keystone directed upwards, as it would be in a reversed arch, this same pressure would displace the uterus downwards.

On this ingenious theory the author briefly bases his treatment. In conjunction with pessaries made according to Hodge's principle, he proposes in the case of displacements, mostly in multiparæ with relaxed abdominal walls, to replace the absent second plane, the tight anterior abdominal wall, by means of a tight, accurately-fitting binder, which in every displacement, and immediately after every delivery, is to be put on and worn until the abdominal wall has again recovered its tone. The relief which at any rate most women with uterine displacements feel in wearing such an abdominal binder, the author explains by pressing up of the uterus according to his theory, not, as hitherto, by the removal of the pressure of the bowels on the irregularly situated uterus.

THE DIAGNOSIS AND TREATMENT OF OBSTETRIC CASES BY EXTERNAL MANIPULATION, BY DR. PAUL MUNDÉ. Second Article. (*American Journal of Obstetrics*, October, 1879.)—We regret much to find that the brief note we added to our short abstract of the first part of this paper in the December retrospect has hurt the author's feelings. We have carefully re-read the paper, and are willing to confess we were somewhat mistaken in our idea of the object aimed at by Dr. Mundé, which we find was less to instruct obstetricians than to spread a knowledge of this useful branch of gynæcology among the profession. As we regard the papers to be extremely well calculated to effect this object, we now include some parts of the first paper not given fully enough in our first reference, along with an abstract of the second part of the paper.—[A. M.] The external method of manipulation is described, for diagnostic purposes, under four heads—inspection, palpation, percussion, and auscultation, and the information gained by each of these is minutely discussed. Under the head of inspection the author especially mentions the appearance, originally pointed out by Bandl, recognizable when there is some obstacle to the passage of the head, as in contracted pelvis, namely, a transverse furrow about midway between the umbilicus and pubis, due to the futile concentric contractions of the uterine body, when the cervix is jammed against the pelvic brim by the presenting part. He says he has himself seen the same phenomenon in excessive pelvic obliquity with consequent anteversion of the uterus. Under palpation Dr. Mundé advises that not the tips of the fingers merely should be used, but that the palmar surfaces of all the fingers should be gently but firmly pressed into the abdominal parietes. By a "pawing" movement we get an idea of the general configuration of the uterus; by pressing deeply behind the uterus from above we ascertain the period of gestation; by moving the hands along side by side we may feel the mobile portions of the fœtus; while by grasping the hypogastric region with the whole hand the presenting part, whether head or breech, is known. External ballottement will determine the amount of mobility of the fœtus. Any abnormality of the uterus is also often easily detected thus, *e.g.*, subperitoneal fibroid tumors, uterus bicornis, etc. The regular intermittent contractions and relaxations of the uterus, described by Braxton Hicks as a sign of pregnancy, is also a late outcome of this practice of palpation. The movements of the fœtus also serve to show the undoubted presence and life of the child, the probable quantity of amniotic fluid, and the approximate size and strength of the child. Rules are also given for guiding us to a knowledge of the presentation in a given case, whether head, breech, or transverse, and how the presence of twins may be predicted. The death of the fœtus, the character of the uterine contractions, hydrocephalus, rupture of the uterus, and extra-uterine fœtation, may all be made known in time for treatment when skill is acquired in the application of the method. From percussion we are enabled only to confirm information obtained by the other modes of procedure. From auscultation important data may be furnished, and of these the one of most value is the pulsation of the fœtal heart, which has been heard in some cases as early as the fourteenth week of utero-gestation. The author recommends the method of immediate auscultation as giving more certain signs of the life or death of the fœtus. Certain important aids to the diagnosis of the position and presentation of the fœtus are also demonstrated to be obtainable from auscultation. In attempting to predict the sex of the fœtus from the number of its heart-beats, the author suggests that we should remember that several modifying influences have to be taken into consideration—as the period since food was taken, the fœtal pulsations being more rapid after meals—and the size of the child—the larger the child the fewer pulsations of its heart, and conversely, whatever be its sex. In a paper by Paul Budin and Chaignot, it is stated that there is no relation whatever between the weight, cardiac pulsations, and sex of the fœtus. The general opinion of the later observers amounts to the possibility of a "shrewd guess" being made as to the sex of a coming child, but nothing more, and it would be unwise to hazard an opinion in any given case. Various opinions regarding the causation of the funic souffle are mentioned, one holding that it is due to pressure on the cord from twisting round the body, another that it is due to organic valvular disease of the fœtal heart, a third that it results from rapid influx of blood from the small arteries into the disproportionately large cavities of the heart, while according to Schroeder it originates in the arteries of the cord at their junction with the body of the fœtus. Another theory would have it generated in the umbilical vein. The practical deduction which the author draws from a consideration of all the possible theories is, that it indicates obstruction in some form to the fœtal circulation. and he calls attention to the necessity of carefully watching both the fœtal heart and the funic souffle, as any irregularity or growing indistinctness of the fœtal heart, coupled with the persistency and increasing force of the souffle, points to some cause endangering the life of the child.

